# Comparative analysis reveals signatures of differentiation amid genomic polymorphism in Lake Malawi cichlids

**DOI:** 10.1186/gb-2008-9-7-r113

**Published:** 2008-07-10

**Authors:** Yong-Hwee E Loh, Lee S Katz, Meryl C Mims, Thomas D Kocher, Soojin V Yi, J Todd Streelman

**Affiliations:** 1 School of Biology, Petit Institute for Bioengineering and Bioscience, Georgia Institute of Technology, 315 Ferst Drive, Atlanta, Georgia 30332, USA; 2 Department of Biology, University of Maryland, College Park, Maryland 20742, USA

## Abstract

**Background:**

Cichlid fish from East Africa are remarkable for phenotypic and behavioral diversity on a backdrop of genomic similarity. In 2006, the Joint Genome Institute completed low coverage survey sequencing of the genomes of five phenotypically and ecologically diverse Lake Malawi species. We report a computational and comparative analysis of these data that provides insight into the mechanisms that make closely related species different from one another.

**Results:**

We produced assemblies for the five species ranging in aggregate length from 68 to 79 megabase pairs, identified putative orthologs for more than 12,000 human genes, and predicted more than 32,000 cross-species single nucleotide polymorphisms (SNPs). Nucleotide diversity was lower than that found among laboratory strains of the zebrafish. We collected around 36,000 genotypes to validate a subset of SNPs within and among populations and across multiple individuals of about 75 Lake Malawi species. Notably, there were no fixed differences observed between focal species nor between major lineages. Roughly 3% to 5% of loci surveyed are statistical outliers for genetic differentiation (F_ST_) within species, between species, and between major lineages. Outliers for F_ST _are candidate genes that may have experienced a history of natural selection in the Malawi lineage.

**Conclusion:**

We present a novel genome sequencing strategy, which is useful when evolutionary diversity is the question of interest. Lake Malawi cichlids are phenotypically and behaviorally diverse, but they appear genetically like a subdivided population. The unique structure of Lake Malawl cichlid genomes should facilitate conceptually new experiments, employing SNPs to identity genotype-phenotype association, using the entire species flock as a mapping panel.

## Abstract 2

Low coverage survey sequencing shows that although Lake Malawi cichlids are phenotypically and behaviorally diverse, they appear genetically like a subdivided population.

## Background

Cichlid fishes from the East African Rift lakes Victoria, Tanganyika, and Malawi represent a preeminent example of replicated and rapid evolutionary radiation [1]. This group of fishes is a significant model of the evolutionary process and the coding of genotype to phenotype, largely because tremendous diversity has evolved in a short period of time among lineages with similar genomes [2-4]. Recently evolved cichlid species segregate ancestral polymorphism [5,6] and may exchange genes [7,8]. Numerous genomic resources have been developed for East African cichlids (many of which are summarized by the Cichlid Genome Consortium [9]). These include the following: genetic linkage maps for tilapia [10-12] and Lake Malawi species [10,13]; fingerprinted bacterial artificial chromosome libraries [14]; expressed sequence tag sequences for Lake Tanganyika and Lake Victoria cichlids [15]; and first-generation microarrays [16,17]. Many studies have used these resources to study cichlid population genetics, molecular ecology, and phylogeny (for review [18,19]). Recent reports have capitalized on the diversity among East African cichlids to study the evolution and genetic basis of many traits, including behavior [20], olfaction [21], pigmentation [22-24], vision [25,26], sex determination [24,27], the brain [28], and craniofacial development [10,13,29].

In 2006, under the auspices of the Community Sequencing Program, the Joint Genome Institute (JGI) completed low coverage survey sequencing of the genomes of five Lake Malawi species. Species were chosen to maximize the morphological, behavioral, and genetic diversity among the Malawi species flock. This represents a novel genome project. Low coverage sequencing is now a routine strategy to uncover functional or 'constrained' genomic elements [30]. The rationale is as follows; one compares genome sequences of distantly related organisms (for example, shark, diverse mammals) with that of a reference (for instance, human, mouse), and outliers of similarity will be observed against the background expectation of divergence [31-34]. Our interests in diversity suggest a conceptually similar but logically reversed research objective. When the background expectation is similarity, how does one use low coverage genome sequencing to detect that which makes organisms distinct?

Here, we report computational and comparative analyses of survey sequence data to address the question of diversity. We had four major goals: to produce a low coverage assembly for each of the five Lake Malawi species; to identify orthologs of vertebrate genes in these data; to predict single nucleotide polymorphisms (SNPs) segregating between species; and to use SNPs to evaluate the degree of genomic polymorphism and divergence at different evolutionary scales. Consequently, we produced assemblies for the five species ranging in aggregate length from 68 to 79 megabases (Mb), identified putative orthologs for more than 12,000 human genes, and predicted more than 32,000 cross-species segregating sites (with about 2,700 located in genic regions). We genotyped a set of these SNPs within and between Lake Malawi cichlid lineages and demonstrate signatures of differentiation on the background of similarity and polymorphism. Our work should facilitate further understanding of evolutionary processes in the species flocks of East African cichlids. Moreover, the approach we outline should be broadly applicable in other lineages where phenotypic and behavioral diversity has evolved in a short window of evolutionary time.

## Results

### Sequence assembly

Trace sequences of five Lake Malawi cichlid species, namely *Mchenga conophorus *(MC; formerly genus *Copadichromis*), *Labeotropheus fuelleborni *(LF), *Melanochromis auratus *(MA), *Maylandia zebra *(MZ; formerly genus *Metriaclima*) and *Rhamphochromis esox *(RE), were downloaded from the GenBank Trace Archive and assembled into contiguous (contig) sequences. The average cichlid genome is 1.1 × 10^9 ^bases [35], so the traces represent a sequence coverage of 12-17% for each of the five species (see Additional data file 1). Through several quality filtering and assembly steps (see Materials and methods [below]), the resultant genomic assemblies of the five cichlid species yielded an average of 60,862 contigs with a mean length of 1,193 bases per contig. The total first-pass assembly sequence length for each species ranged from 68,238,634 bases (MA) to 79,168,277 bases (MZ), or about 7% of an average cichlid genome. Assembly statistics are shown in Table 1.

**Table 1:** First-pass genomic assembly statistics for five Lake Malawi cichlid species

	MC	LF	MA	MZ	RE
Total number of contigs in assembly	61,923	58,245	63,297	65,094	55,751
Total length (bases)	73,425,564	70,858,381	68,238,634	79,168,277	71,295,074
Genome coverage^a ^(%)	6.68	6.44	6.20	7.20	6.48
Mean trace length (bases)	1,055	1,092	991	1,145	1,153
Shortest contig length (bases)	50	50	50	50	50
Longest contig length (bases)	19,632	17,437	21,601	15,371	21,351
Mean contig length (bases)	1,186	1,217	1,078	1,216	1,279
Q25 contig length (bases)	759	846	783	805	934
Q50 (median) contig length (bases)	966	1,063	949	1,163	1,113
Q75 contig length (bases)	1,403	1,355	1,102	1,417	1,407

Total genic length (bases)	2,863,110 (3.9%)	2,841,933 (4.0%)	2,761,941 (4.0%)	2,851,968 (3.6%)	2,797,548 (3.9%)

^a^Using an average cichlid genome size of 1.1 × 10^9 ^bases. LF, *Labeotropheus fuelleborni*; MA, *Melanochromis auratus*; MC, *Mchenga conophorus*; MZ, *Maylandia zebra*; RE, *Rhamphochromis esox*; Q25, 25^th ^percentile; Q50, median or 50^th ^percentile; Q75, 75^th ^percentile.

We noted that these first-pass assemblies were 'over-assembled' by roughly a factor of 2 when compared with theoretical expectations [36]. Theory suggests that random shotgun sequencing of single copy DNA, at 15% coverage of a 1.1 gigabase genome, will result in an assembly length of about 153 Mb. We reasoned that our assemblies might be shorter than expected because multicopy elements were grouped as if they were single copy sequence. Given the theoretical expectation (again for 15% coverage of a 1.1 gigabase genome) that individual bases should only be sequenced a maximum of four to five times, we examined whether contigs were built from five or more trace sequences contributing overlapping bases. We observed that about 10 Mb of each first-pass assembly were derived from such contigs, and excluded these data from subsequent analyses (for example SNP prediction [see below]). Notably, individual sequences contributing to these 'high trace number' contigs were not identified by RepeatMasker but did sometimes have Basic Local Alignment Search Tool (BLAST) matches to putative repetitive elements (for example, pol polyprotein, reverse transcriptase). Because of the keen interest in repetitive DNA families in cichlids [37] and other organisms [38], we have retained alignments of these 'high trace number' contigs and have marked them as such (see Additional data files 3 and 4).

### Gene content and coverage

To establish the extent of gene content and coverage present in each assembly, we carried out BLASTX similarity searches (10^-10 ^E value cutoff) for each of the five assemblies against a reference human proteome (RefSeq proteins). The average proportion of putative genic sequence amounted to 3.9% of the available genomes. The MZ assembly contained the highest gene coverage, possessing genic loci that were significantly similar to approximately 5,240 unique human proteins. The remaining four species yielded approximately similar numbers ranging from 5,020 to 5,170 genes. It must be noted, however, that most of these genes are highly fragmented and incomplete, because of low coverage of the assembly. In all, a total of 36% (12,211 genes out of 34,180; see Additional data file 2) of the reference human proteome could be identified in one or more of the cichlid species.

### Clustering and alignment

**Figure 1:**
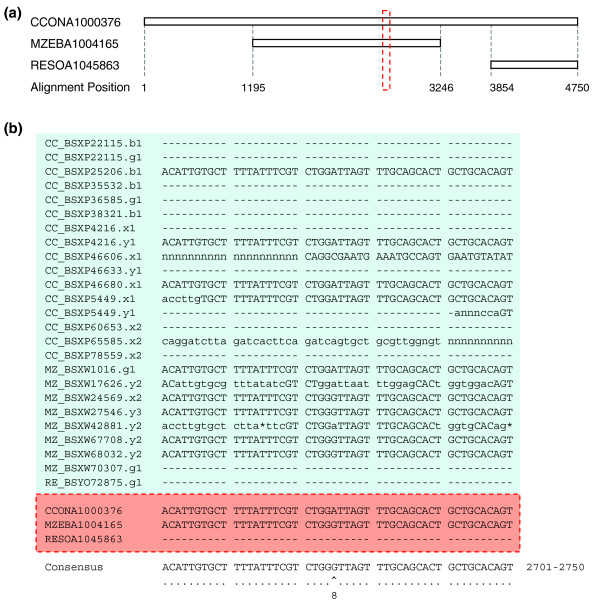
Alignment of a typical cluster of orthologous sequences. **(a) **Overall alignment of assembly contigs from three different cichlid species with alignment positions indicated. **(b) **Expanded detail of nucleotide alignment. Filled pink block shows the expanded alignment corresponding to dotted red box in panel a. Filled blue block shows the alignment of corresponding species' traces that made up the assembly sequences. Lower case nucleotides have base quality scores under 20. Dashes '-' represent sequence unavailability. Asterisks '*' represent gaps inserted into the sequences. Dots '·' represent identity in alignment. Cap '^' represents segregating site. Alignment positions shown after consensus sequence. Polymorphism quality score shown below A-G single nucleotide polymorphism site.

We obtained 25,458 clusters of putatively orthologous sequences, which were individually assembled into multi-species alignments for subsequent comparative analyses. Genic regions, as identified by similarity searches to known human and fish genes, were marked onto each alignment. Figure 1 illustrates a typical example of one such alignment.

Roughly 1% of the alignments (294 alignments) showed percentages of variable sites above 2% (about tenfold higher than the average). It is impossible to know, given the low coverage of the sequenced genomes, whether these represent orthologous but divergent regions of cichlid genomes or the alignment of paralogous sequence. We therefore retained these alignments, and included a calculation of polymorphism for each alignment (see Additional data file 3), for the consideration of researchers using these data. For example, alignment 108,866 contains sequence with similarity to asteroid homolog 1, with 8% of sites variable and a majority of replacement polymorphism. Given the lack of functional information about this novel signaling protein (first described in *Drosophila *[39]), this alignment provides useful information even if (and perhaps because) it includes paralogous loci. Another 12% of the alignments (2,119 total) contained individual species contigs that had consensus base positions derived from five or more trace sequences (see above).

For all subsequent analyses, we excluded 2,413 alignments that exhibited a high percentage of variable sites and/or higher than expected coverage. More than 11.6 million bases of multiple species alignments remain, of which roughly 1.06 Mb were inferred as genic. This included 10,902,011 (986,506 genic) bases of two-species alignments, 721,049 (75,371 genic) bases of three-species alignments, 27,951 (2,898 genic) bases of four-species alignments, and 877 (193 genic) bases of alignments containing all five species.

### Segregating sites

Further analysis of these 11.6 million bases of multiple alignments identified a total of 32,417 (0.28%) cross-species SNPs. In order to classify the quality of an identified variable site, a polymorphism quality score (PQS) was defined, corresponding to the first digit of the lowest Phrap quality score among the nucleotides of the different species present at the polymorphic site (for example, a polymorphic site between four species with base quality scores of 34, 45, 46, and 50 would be assigned a PQS of 3). In total, 4,468 (13.8%) variable sites had a PQS of 5 or higher, 7,952 (24.5%) had a PQS of 4, 8,236 (25.4%) a PQS of 3, and the remaining 11,761 (36.3%) had a PQS of 2. PQS for each variable site are provided on the alignments described in Additional data file 3 (also available online [40]). Nucleotide diversity (Watterson's θ_w_) averaged over two-, three-, and four-species alignments was 0.00257. Roughly 8% of all polymorphic sites (2,709) were located within the putative genic regions identified earlier. Alignments with fish and human proteins provided us with the phase information required to further classify these into 1,066 synonymous and 1,643 nonsynonymous SNPs. Summaries of all alignments containing genic and nongenic polymorphisms are provided in Additional data files 3 and 4.

In order to investigate the pair-wise differences between any two of the five species, all sequence alignment segments with two or more species were broken up into all possible pair-wise alignments; this resulted in 1.06 to 1.55 Mb of alignment per pair. We then calculated the Jukes-Cantor distance between species pairs. The three shortest distances were between LF and MZ (0.229%), followed by MA/MZ (0.232%) and LF/MA (0.241%), and the greatest was between LF and RE (0.288%). These genetic distances include both within-species polymorphism and the fixed differences between species. Currently, there is no exhaustive estimate of within-species polymorphism for Malawi cichlids. Unpublished data from our own group (Streelman JT) indicates that for LF and MZ, within-species diversity (π) may be as high as 0.2%. Thus, the percentage of fixed genetic differences is likely to be extremely small in this assemblage (see following sections).

Finally, we calculated the ratio of replacement to synonymous substitutions (K_a_/K_s_) for concatenated genic alignments among all pairs of species. We used concatenated sequences because each segment represented only a small fraction of a gene, with only few nonsynonymous and synonymous sites. K_a_/K_s _ranged from 0.380 in MC/LF to 0.562 in LF/MA. These numbers are greater than the ratios found between *Fugu *and *Tetraodon *(0.127 to 0.144 [41]). Such high K_a_/K_s _values may indicate that positive selection, driven by adaptive radiation, is prevalent in cichlid fishes. However, given the expectation of few fixed differences between groups, this topic should be revisited with more data on the levels of segregating and fixed nucleotide substitutions among lineages.

### Validation and generality of SNPs

We genotyped 96 SNPs in 384 Lake Malawi cichlid samples using Beckman Coulter SNPstream™ technology (Beckman Coulter, Inc., Fullerton, CA). The SNPs were partitioned into three categories to help us evaluate the comparative success rate of automated SNP prediction. First, we included 13 positive controls: genes previously sequenced by others [3,25] and by us (Streelman JT, unpublished data), with expected variation in Malawi cichlids. Positive controls included genes involved in morphogenesis (*otx1*, *otx2*, and *pax9*), pigmentation (*mitf*, *ednrb*, and *aim1*), and visual sensitivity (opsins *rh1*, *sws1*, *lws*, *sws2a*, and *sws2b*). Next, we genotyped 59 SNPs identified using the automated procedure described in this report. We selected these SNPs to represent a range of PQS (from 2 to 5) and a variety of sequence types (genic, nongenic with a BLAST match < e^-100 ^to *Tetraodon*, and nongenic with no BLAST match). Finally, we wished to compare our automated SNP selection to a manual approach. Therefore, we included an additional 24 SNPs identified by manual inspection of BLAST matches between single JGI traces and *Tetraodon *chromosome 11; we have previously shown *Tetraodon *11 to share orthologs with cichlid chromosome 5 [13]. Note that these SNPs were most often not discovered by our automated procedure because they originated in single traces that did not meet percentage quality cutoffs and/or they did not align into comparative contigs because of overlap cutoffs.

Our validation strategy sought to document the general use and segregation of these markers among Lake Malawi cichlids. Given recent divergence times among species (some as recent as 1,000 years [2]), we expected that SNPs might segregate throughout the assemblage. Therefore, Malawi samples comprised about ten individuals from each of ten populations of MZ and LF, as well as one to five individuals of 77 additional species (25 of which were rock-dwelling mbuna). Taxa were included to represent the morphological, functional, and behavioral diversity of the Malawi lineage, which may contain more than 800 species [42].

Ten out of 13 (about 77%) positive controls gave reliable genotypes and were variable across the dataset. For the 59 SNPs predicted by our automated procedure, 11 were fixed (no variation) in all samples, indicating an error in sequencing (or genotyping), an error in prediction, or the presence of a low frequency allele in the sequenced samples. Six predicted SNPs did not produce data reliable enough for genotype calls. The remaining 42 loci from automated predictions (about 71%) were polymorphic across the dataset. For 24 SNPs predicted using manual similarity searches, four were fixed and four failed reliability for genotype calls, with the remaining 16 loci (about 67%) showing polymorphism (Table 2). Twelve out of 20 (60%) predicted SNPs with PQS of 3 or less were successful, whereas 30 out of 39 (76%) predictions with PQS of at least 4 yielded polymorphisms (Table 3). There is evidence of ascertainment bias in our genotypic data (see Additional data file 5). For example, three SNP loci (Aln100674, Aln114498, and Aln102321) exhibit alleles unique to *Rhamphochromis*. Similarly, SNPs predicted from comparisons of RE and mbuna (LF, MA, and MZ) are sometimes fixed in mbuna. Polymorphisms predicted from comparisons of mbuna taxa are more likely to vary within LF and MZ populations and across mbuna species.

**Table 2:** SNP genotyping success categorized by detection method

SNP detection method	Control genes	Automated	Manual BLAST
Number of genotyped loci	13	59	24
Number of polymorphic loci	10	42	16
Number of fixed loci	3	11	4
Number of failed loci	0	6	4

Successful SNP detection (%)	76.9	71.2	66.7

BLAST, Basic Local Alignment Search Tool; SNP, single nucleotide polymorphism.

**Table 3:** SNP genotyping success categorized by polymorphic quality score

Polymorphic quality score	2	3	4	5
Number of genotyped loci	5	15	28	11
Number of polymorphic loci	2	10	24	6
Number of fixed/failed loci	3	5	4	5

Successful SNP detection (%)	40	66.7	85.7	54.5

SNP, single nucleotide polymorphism.

### Genetic polymorphism and divergence at multiple scales

Strikingly, among all 68 loci showing polymorphism, no SNP locus was alternately fixed between LF and MZ, or between rock-dwelling mbuna and non-mbuna. We thus sought to investigate the degree of polymorphism versus divergence at multiple evolutionary scales.

**Figure 2:**
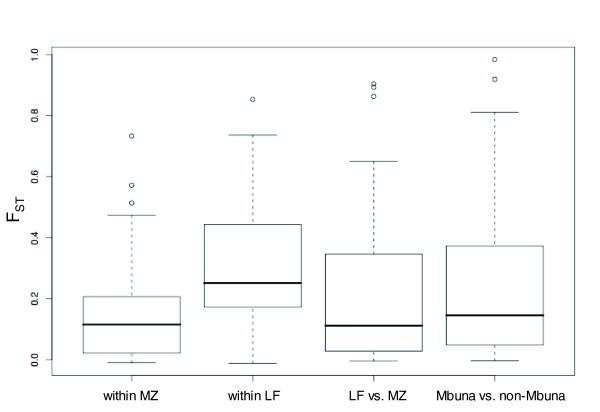
Box-and-whisker plots of F_ST _values. F_ST _values were calculated for the following: within MZ, within LF, LF versus MZ, and Mbuna versus non-Mbuna. Upper and lower box bounds represent 75th and 25th percentiles, respectively. The solid lines within boxes represent the median value. Whiskers mark the furthest points from the median that are not classified as outliers. Unfilled circles represent outliers that are more than 1.5 times the interquartile range higher than the upper box bound. F_ST_, genetic differentiation; LF, *Labeotropheus fuelleborni*; MA, *Melanochromis auratus*; Mb, megabases; MC, *Mchenga conophorus*; MZ, *Maylandia zebra*.

The data (Additional data file 5) support the previously reported population structures in MZ [43,44] and LF [45], as well as the genetic distinction between these species (MC Mims, unpublished data). For example, mean genetic differentiation (F_ST_) in MZ is 0.148 and in LF is 0.271. Mean F_ST _between LF and MZ was 0.215, and between mbuna (25 species) and non-mbuna (52 species) it was 0.224, demonstrating that most genetic variation segregates within and not between lineages, regardless of evolutionary scale. Nevertheless, these distributions of F_ST _yielded statistical outliers, which exhibit greater than average genetic differentiation (Figure 2). Four loci were found to be statistical outliers for F_ST _among MZ and LF populations. In MZ the opsin loci *lws *(F_ST _= 0.514), *sws1 *(0.572) and *rh1 *(0.733), and in LF the opsin locus *rh1 *(0.853) exhibit differentiation between populations. Between LF and MZ, three loci were identified as outliers: a nonsynonymous polymorphism in *csrp1 *(F_ST _= 0.893), a synonymous polymorphism in *β-catenin *(Aln101106_1089; F_ST _= 0.904), and an intronic polymorphism in *ptc2 *(Aln100281_1741; F_ST _= 0.863). Two statistical outliers were identified for F_ST _between rock-dwelling mbuna and non-mbuna groups: a nonsynonymous polymorphism in *irx1 *(Aln102504_1609; F_ST _= 0.984), and a nongenic polymorphism (Aln103534_280; F_ST _= 0.919) in sequence with similarity to pufferfish and stickleback genomes between *contactin 3 *and *ncam L1*.

### Genetic clustering and ancestry

**Figure 3:**
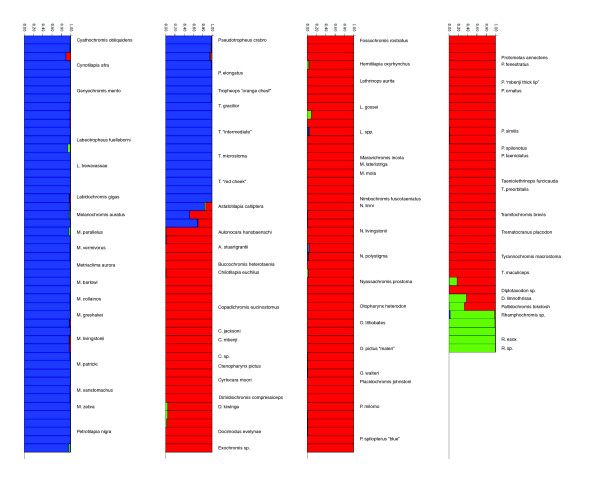
Bayesian assignment of Lake Malawi cichlids to different evolutionary lineages. We show the contribution to each individual genome (q, which ranges from 0% to 100%) from each of K = 3 predefined genetic clusters (blue, red, and green), for data derived from single nucleotide polymorphisms (SNPs) in Tables 2 and 3. Note that this method predefines the number but not the identity of genetic clusters. Species names are written once; multiple individuals from species are grouped together (for example, four individuals of *Pseudotropheus crabro*). Species considered mbuna (blue) cluster with other mbuna, to the exclusion of other groups; species thought to represent the earliest divergence within the species flock (*Rhamphochromis*) clustered together as a separate group (green); and all remaining non-mbuna species formed the third group (red).

To further visualize the segregation of SNPs across the Malawi cichlid flock, we utilized a Bayesian approach that assigns individuals to a predefined number of genetic clusters [46]. Specifically, we were interested in how species would be assigned to major Malawi cichlid lineages identified in previous studies [3,4,47]. There are three such groups supported by the majority of molecular data: the rock-dwelling mbuna; pelagic and sand-dwelling species; and a group comprised of *Rhamphochromis*, *Diplotaxodon*, and other deep-water taxa. Analysis of 68 SNP loci accurately classifies species to respective lineages (Figure 3). For instance, all species considered mbuna (blue) cluster with other mbuna, to the exclusion of other groups; species thought to represent the earliest divergence within the species flock (*Rhamphochromis*) clustered together as a separate group (green); all remaining non-mbuna species formed the third group (red). Notably, deepwater genera *Diplotaxodon *and *Pallidochromis *contain individuals with mosaic genomes (red and green) and *Astatotilapia calliptera*, a nonendemic species and possible Malawi ancestor [48] combines mbuna and non-mbuna genomes.

For comparison, additional analyses were performed setting the predefined number of genetic clusters to from two to five. When set to two genetic clusters, species were accurately classified as mbuna or non-mbuna. At settings of four or five, the program was unable to yield stable classification results between replicate runs. Thus, these latter three sets of analyses (data not shown) did not provide any further insights into the genetic lineages of Malawi cichlids.

## Discussion

African cichlid fishes are important models of evolutionary diversification in form and function [44]. They are singularly remarkable for the extent of phenotypic and behavioral diversity on a backdrop of genomic similarity. Lake Malawi is home to the most species-rich assemblage of African cichlids; as many as 800 to 1,000 species are thought to have evolved from a common ancestor during the past 500,000 to 1 million years ago [42]. These recently formed species segregate ancestral polymorphism and exchange genes by hybridization [5,7,49]. Such circumstances present both opportunities and challenges for understanding evolutionary history and biological diversity. Opportunistically, researchers have used molecular markers across studies to interrogate the genetic basis of phenotypic differentiation [13,22,24,29]. This approach views Malawi cichlid species as natural mutants screened for function by natural selection, with essentially identical ancestral genomes honed by contrasting historical processes. By contrast, the task of reconstructing a phylogeny of species has been hindered by the very same phenomena of genomic similarity and mosaicism [2,3]; even the promising approach of Amplified Fragment Length Polymorphism (AFLP) does not provide strong resolution of the relationships among genera [23,48,50,51]. The data we present here should provide new resources and perspectives for cichlid evolutionary genomics.

### Cichlid species exhibit genomic polymorphism

Lake Malawi cichlid species sequenced by the JGI embody the phylogenetic, morphological, and behavioral diversity found within the assemblage. *Rhamphochromis esox *(RE) is a large (about 0.5 m) pelagic predator that represents one of the basal lineages of the species flock [3,4,47]. *Mchenga conophorus *(MC) is a sand-dwelling species that breeds on leks, where males construct 'bowers' to attract females. *Melanochromis auratus *(MA), *Maylandia zebra *(MZ), and *Labeotropheus fuelleborni *(LF) are rock-dwelling (mbuna) species that differ in color pattern, trophic ecology, body shape, and craniofacial morphology (pictures of these and others are available online [52]).

Our data confirm the conclusions from previous genetic analyses on a smaller scale; Lake Malawi species are genetically similar. Nucleotide diversity observed among the five cichlid species (Watterson's θ_w _= 0.26%) is less than that found among laboratory strains of the zebrafish *Danio rerio *(Watterson's θ_w _= 0.48% [53]). Although overall nucleotide diversity is less than that observed in *Danio*, the ratio of replacement to silent change is nearly fivefold higher in the Lake Malawi genomes. Such a result might suggest that East African cichlid evolution is characterized by adaptive molecular evolution, as has been indicated in a few instances [25,54], or a relaxation of purifying selection attributable to small effective population size. However, we should view this estimate of K_a_/K_s _with caution because of one of the remarkable features of these data (see below). Variable sites identified from cross-species alignments are not substitutions fixed between species. The K_a_/K_s _approach to identifying selection may be largely inappropriate for such young species where ancestral alleles segregate as polymorphisms.

The pattern of variation observed across the approximately 75 species genotyped in this study demonstrates that biallelic polymorphisms segregate widely throughout the Malawi species flock. SNPs segregate within and between MZ and LF populations, as well as within and among mbuna species and other lineages. No SNP locus surveyed is alternately fixed in LF versus MZ, nor between mbuna and non-mbuna. Remarkably, the degree of genetic differentiation (F_ST_) within species is roughly equivalent to that between species and to that between major lineages. Lake Malawi cichlid species are mosaics of ancestrally polymorphic genomes. Add to this a propensity of recently diverged species to exchange genes [2], and Malawi cichlids present a case of complex and dynamic evolutionary diversification, where recombination and the sorting of ancestral polymorphism may be more important than new mutation as sources of genetic variation. Despite allele sharing, SNP frequencies contain a clear signal of ancestry for the entire flock. Rock-dwelling mbuna comprise a genetic cluster, as do pelagic and sand-dwelling species, in addition to *Rhamphochromis*. Notably, *Astatotilapia calliptera*, one of a few nonendemic haplochromines in Lake Malawi, appears to retain a reservoir of ancestral polymorphisms from which mbuna and non-mbuna genomes have emerged.

### Genomic polymorphism and the divergence of Malawi cichlids

Our hierarchical sampling design allows us to consider whether there are loci exhibiting extreme genetic differentiation against the background of shared polymorphism within species, between species, and between major lineages. Strikingly, regardless of the evolutionary scale, statistical outliers comprise approximately 3% to 5% of loci surveyed. Opsin loci *lws*, *rh1*, and *sws1 *are differentiated among populations of LF and MZ, adding to reports that opsin polymorphisms are associated with population-specific color patterns or visual environments [55].

SNPs in *csrp1*, *β-catenin*, and *ptc2 *exhibit greater than expected differentiation between LF and MZ. *Csrp1 *(cysteine-rich protein) is a vertebrate LIM-domain family member acting in the noncanonical WNT pathway, expressed in gut, intestine, and cardiac mesoderm [56]. *β-catenin *acts to transduce signals in the canonical WNT pathway [57] and is expressed in developing cichlid fins, dentitions, brains, and lateral lines (Fraser GJ, Streelman JT, unpublished data). Patched is a receptor for sonic hedgehog [58]; both areexpressed in developing cichlid dentitions, jaws, and brains (Fraser GJ, Sylvester JB, Streelman JT, unpublished data). A SNP in *irx1 *nearly perfectly differentiates rock-dwelling mbuna from the remainder of the Malawi species flock. *Irx1 *acts to position the boundary between the telencephalon and the posterior forebrain [59]. Finally, a SNP located between *contactin 3 *and *ncam L1 *exhibits differentiation between mbuna and non-mbuna lineages; these genes are linked in other genomes and functionally interact to pattern dendritic branching in the neocortex [60]. Taken together, differentiated loci are interesting in the context of cichlid diversification because they affect the phenotypes that vary among lineages: color and vision [25,26], guts [61], dentitions [13,62], jaws [10,29], and brains [28].

### Discovery for evolutionary biology

There are obvious challenges when attempting to extract information from low coverage genomic sequence, and also obvious payoffs [31-34]. Most previous studies have used this information for species-specific discovery (for example, dog breeds) or broad evolutionary comparisons with respect to a reference genome (for example, dog-human, shark-human, or cat-mammal). Our goals in the present analysis stem from the unique characteristics of Lake Malawi cichlids; these are biologic species that behave genetically like a single subdivided population. Therefore, our biggest challenge was to devise a strategy that retains information from these low coverage survey sequences (75% genomic coverage spread over five closely related species), but minimizes error and bias in assembly and cross-species alignment for SNP identification. For example, we excluded many contigs because they appeared to be over-assembled, and we excluded multi-species alignments if they exceeded a polymorphism threshold. The over-assembly problem limits the coverage of these genomes in relation to expectation; this phenomenon, observed in the cat genome and in simulation, has complex and varying causes and has yet to be fully resolved [63]. It is likely to be mitigated to some degree by comparison with a higher coverage reference sequence. The power of the data we present comes from the broad utility of the genic sequences and SNPs we have identified for many questions in genomic evolutionary biology.

Our analyses identified about 12,000 Lake Malawi cichlid sequences with similarity to human and fish proteins. This is a significant advance in our understanding of cichlid genomic content. To put this in context, approximately 13,500 unique expressed sequence tags, from three different East African cichlids, represent the sum total of such publicly released sequences [15]. Our contribution roughly doubles the available data.

The approximately 32,000 (2,700 genic) SNPs we identified should provide a wealth of molecular markers for studies of population genetics and molecular ecology, linkage and quantitative trait locus mapping, association mapping, and phylogeny. We convert about 70% of predicted SNPs to polymorphic markers; this percentage is comparable to that of other studies from white spruce (74% to 85%, depending on quality cutoffs [64]), zebrafish (65% [53]), and cow (43% [65]). We have shown these biallelic markers to be of general use, many segregating across the major cichlid lineages of Lake Malawi. We used the SNPs to assign Malawi species to ancestral genetic clusters, and this approach should hold promise for similar questions of genetic structure that span the population versus species continuum. It is important to note that early runs of this analysis, with fewer SNP loci, resulted in stable results with more individuals showing mosaic genomes. This suggests that careful consideration should be given to the number of polymorphic loci necessary to yield confidence in evolutionary interpretation. As more SNP loci (with known genome coordinates) are assayed, it will be possible to compute and compare ancestry proportions across scales (for example, genome versus chromosome versus gene cluster).

Notably, we have used the background level of genomic similarity and polymorphism to identify loci that may have experienced a history of selection within species, between species and between major lineages. Because SNP markers are co-dominant, easy to genotype, reliable and reproducible from laboratory to laboratory, and readily mapped in silico (NHGRI will sequence a related cichlid, the tilapia, to 7-fold draft assembly coverage in 2008), they are likely to complement microsatellites and AFLP for most applications in cichlid evolutionary genomics. Given the unique mosaic structure of Lake Malawl cichlid genomes, it is exciting to envision experiments employing SNPs to identity genotype-phenotype associations, using the entire species flock as a mapping panel. Finally, as sequencing costs continue to drop, the approach we outline here should prove applicable to those studying evolutionary and phenotypic diversity among closely related species [44].

## Materials and methods

### Samples

Individuals of *Mchenga conophorus *(MC), *Labeotropheus fuelleborni *(LF), *Melanochromis auratus *(MA), *Maylandia zebra *(MZ), and *Rhamphochromis esox *(RE) were sampled from the wild during an expedition to Malawi in 2005. Specimens prepared for survey sequencing by the JGI were collected from Mazinzi Reef (MZ), Domwe Island (LF and MA), and Otter Point (MC and RE), all of which are locales in the southeastern portion of the lake. High-quality DNA was extracted and prepared in the laboratory of TDK.

### Trace sequences

Trace sequences generated by the JGI for MC, LF, MA, MZ, and RE, together with their sequence quality scores, were downloaded (6 May 2007) from the National Center for Biotechnology Information (NCBI) Trace Archive. The dataset for each species consisted of an average of about 152,000 individual trace reads with total read lengths ranging from 137 to 185 million bases. Detailed sequence statistics for each species are provided in Additional data file 1.

### Sequence preprocessing and assembly

The trace and quality sequences were first pre-processed for assembly by masking out all possible vector sequences available from the NCBI UniVec vector sequence database (downloaded 6 May 2007). The vector masking was performed using the cross_match.pl perl script provided by the Phred-Phrap package [66]. In order to reduce the computational complexity and time required for the final assembly, repeat sequences were masked before assembly using RepeatMasker version 3.1.8 (Smit AFA, Hubley R and Green P, unpublished data) in conjunction with the latest repeatmasker libraries from RepBase Update [67]. Bases with sequencing quality score of less than 20 were also masked. The actual assembly of each species' trace sequences into contiguous sequences (contigs) was then performed using the Phrap version 0.990329 assembly program from the Phred-Phrap package. Contigs with more than 80% low quality bases (defined as <20 assembly quality score) were removed from the assembly. This whole genome shotgun project has been deposited at DDBJ/EMBL/GenBank under the project accessions ABPJ00000000 (MC), ABPK00000000 (LF), ABPL00000000 (MA), ABPM00000000 (MZ), and ABPN00000000 (RE). The versions described in this paper are the first versions: ABPJ01000000, ABPK01000000, ABPL01000000, ABPM01000000, and ABPN01000000.

### Similarity search and alignment

Orthologous genomic contig pairs were first identified using reciprocal BLASTN similarity searches with a strict E-value cutoff of 10^-100^, performed across the sequence contigs of all possible species pairs. To reduce spurious ortholog assignments, putative ortholog contig pairs were only retained if their regions of high sequence similarity formed good end-to-end overlaps (defined as within 100 bases of the 5' end or 30 bases from the 3' end of a sequence) or overlap more than 80% of the shorter contig. Although some of the filtered regions could represent biologically relevant loci where recombination or translocations might have occurred, we decided to remove them from this analysis. Contig pair assignments were then passed to an algorithm that created clusters of contigs whereby each contig within the cluster must be related to all other contigs in the cluster through one or more putatively orthologous relations.

Each cluster of contigs was then individually aligned using Phrap, resulting in a continuous alignment tiling path where each alignment position may consist of a base from any one or up to all five cichlid species (Figure 1). Segregating sites were then identified from alignment positions with high quality bases (>20 score) from two or more species. A PQS was defined, corresponding to the first digit of the lowest Phrap quality score among the nucleotides of the different species present at the polymorphic site (for example, a polymorphic site between four species with base quality scores of 34, 45, 46, and 50 would be assigned a PQS of 3). To compare the extent of nucleotide diversity among the five cichlid species, we calculated Watterson's theta (θ_w _[68]). This measure takes into account the number of variable positions and the sample size analyzed. Our data violate the assumption of an infinite, interbreeding population, but we chose this metric to in order to make direct comparisons to similar measures from study of other genomes (for example, zebrafish).

### Protein-coding sequence identification

Cichlid protein coding sequences were inferred based on similarity searches to known protein databases of fishes and humans. BLASTX searches with E-value cutoff of 10^-10 ^were performed for the each cichlid genomic assembly as well as the overall consensus sequence of the cluster alignments, against a protein database made up of all GenBank *Actinopterygii *(ray-finned fishes) sequences (downloaded 2 June 2007; 163,471 entries) and all human RefSeq proteins (downloaded 25 June 2007; 34,180 sequences). The alignment with the highest scoring hit for each genomic locus was then used as a reference to determine the coding strand and phase of the protein-coding cichlid locus.

### Evolutionary sequence divergence among JGI species

All cluster alignment segments with contributing bases from two or more species were split into pairwise alignments (each two, three, four, or five species alignment position can be split into one, three, six, or ten pair-wise alignments respectively). Pair-wise alignments within each of the ten possible species pair combinations (MC-LF, MC-MA, MC-MZ, MC-RE, LF-MA, LF-MZ, LF-RE, MA-MZ, MA-RE, and MZ-RE) were then concatenated and the number of substitutions counted. Jukes-Cantor correction for multiple substitutions was applied to these direct distance measurements [69]. Pair-wise alignments consisting of only genic sequences were obtained from multi-species cluster alignment segments in a manner similar to that described above. The DNAStatistics package of Bioperl [70] was then used to calculate the K_a_/K_s _values of pair-wise alignments.

### Genotyping and validation of SNPs

We genotyped 96 SNPs in 364 diverse Lake Malawi cichlid samples. These SNPs included 13 positive controls, 59 loci from the automated procedure described in this report, and an additional 24 loci chosen manually by BLAST of individual traces to the *Tetraodon *genome (see main text for further description). The GenomeLab SNPstream Genotyping System Software Suite v2.3 (Beckman Coulter, Inc.) was used for experimental setup, data uploading, image analysis, genotype calling and QC review, at Emory University's Center for Medical Genomics. In brief, marker panel data (multiplexed SNP panel designed by SNPstream's Primer Design Engine website [71]) were first uploaded to the SNPstream database using the PlateExplorer application software. Also uploaded was the Process Group Data containing all test sample information generated through a Laboratory Information Management System (Nautilus 2002; Thermo Fisher Scientific, Waltham, MA, USA). An on-board CCD camera of the SNPstream Imager took two snapshot images of each well of the 384-well tag array, one under a blue excitation laser and the other under a green excitation laser. Image application software was used to analyze the captured images to detect spots, overlay an alignment grid, and determine spot intensity. The fluorescent pixel intensity data for each SNP under the two channels, representing the relative abundance of the two alleles, were uploaded to the database. The GetGenos application software was used to calculate and generate a Log(B+G) versus B/(B+G) plot, where B and G were the pixel intensities under the blue and green channels, respectively, for each sample and each SNP. Next, automated genotype calling was accomplished using the QCReview application software based on a number of criteria (for instance, signal baseline, clustering pattern of the three genotypes, and Hardy-Weinberg score). A genotype summary was generated using the Report application software.

### Genetic differentiation within and among lineages

Locus-specific F_ST _[72] was calculated using FSTAT version 2.9.3.2 [73] for three evolutionary scales: within LF and MZ; between LF and MZ; and between mbuna and non-mbuna. We determined that a SNP locus was a statistical outlier using the empirical distribution of F_ST _values. F_ST _outliers exceed the sum of the upper quartile value and 1.5 times the interquartile range.

### Genomic assignment

We used a Bayesian method (STRUCTURE v.2.2 [46]) to determine how well our SNP genotypes assigned individuals to evolutionary lineages. We chose to define the number of K genetic clusters in accord with previous research showing about three major evolutionary groups of Lake Malawi cichlids [3-5,47]. Note that we do not intend this to mean that three is the best supported estimate of K in these data; our rationale is rather to demonstrate how individual genomes are composites (or not) of the major evolutionary lineages found in the lake. Thus, we used the admixture model to estimate q, the proportion of each genome derived from each of K genetic clusters. For comparison, we also ran analyses with K set to two, four, or five (not shown). Each run of the program included 50,000 cycles of burn-in and run length of 50,000 steps. Multiple runs were conducted to ensure reliability and consistency of results.

## Abbreviations

BLAST, Basic Local Alignment Search Tool; F_ST_, genetic differentiation; JGI, Joint Genome Institute; K_a_/K_s_, ratio of replacement to synonymous substitutions; LF, *Labeotropheus fuelleborni*; MA, *Melanochromis auratus*; Mb, megabases; MC, *Mchenga conophorus*; MZ, *Maylandia zebra*; NCBI, National Center for Biotechnology Information; PQS, polymorphism quality score; RE, *Rhamphochromis esox*; SNP, single nucleotide polymorphism.

## Authors' contributions

YHL, JTS, SVY, and TDK conceived the idea and designed the study. YHL, LSK, and MCM performed the research. YHL and JTS analyzed the data and drafted the manuscript. All authors read and approved the final manuscript.

## Additional data files

The following additional data are available with the online version of this paper. Additional data file 1 is a table of trace sequence statistics of five Lake Malawi cichlid species. Additional data file 2 is a list of human gene homologs found in the five cichlid species. Additional data file 3 is a list of alignments and polymorphic sites. Additional data file 4 is a list of alignments with BLAST hits to fish and humans. Additional data file 5 is a table of major allele frequencies for biallelic SNPs surveyed across Lake Malawi cichlid populations and species.

## Supplementary Material

Additional data file 1 Presented is a table of trace sequence statistics of five Lake Malawi cichlid species. Click here for file

Additional data file 2 Presented is a list of human gene homologs found in the five cichlid species. Click here for file

Additional data file 3 Presented is a list of alignments and polymorphic sites. Click here for file

Additional data file 4 Presented is a list of alignments with BLAST hits to fish and humans. Click here for file

Additional data file 5 Presented is a table of major allele frequencies for biallelic SNPs surveyed across Lake Malawi cichlid populations and species. Click here for file
