# Supplementary material for: Comparative analysis reveals signatures of differentiation amid genomic polymorphism in Lake Malawi cichlids
Source: Genome Biol. 2008 Jul 10;9(7):R113. doi: 10.1186/gb-2008-9-7-r113 (PMC2530870; doi:10.1186/gb-2008-9-7-r113)
Supplement: Additional data file 1 — Presented is a table of trace sequence statistics of five Lake Malawi cichlid species. [file gb-2008-9-7-r113-S1.pdf]

Supplementary Table 1. Trace sequence statistics of five Lake Malawi cichlid species.

	C. conophorus	L. fuelleborni	M. auratus	M. zebra	R. esox
Number of trace reads	157,434	153,061	138,517	161,413	152,385
Total read length (bases)	166,071,742	167,074,220	137,257,743	184,775,275	175,769,721
Shortest read length (bases)	72	88	76	109	76
Longest read length (bases)	6,759	7,264	4,862	7,072	5,834
Mean read length (bases)	1,055	1,092	991	1,145	1,153
Q25 read length (bases)	800	893	822	844	976
Q50 (median) read length (bases)	1,040	1,092	995	1,223	1,133
Q75 read length (bases)	1,313	1,237	1,126	1,417	1,383
