# Supplementary material for: Comparative analysis reveals signatures of differentiation amid genomic polymorphism in Lake Malawi cichlids
Source: Genome Biol. 2008 Jul 10;9(7):R113. doi: 10.1186/gb-2008-9-7-r113 (PMC2530870; doi:10.1186/gb-2008-9-7-r113)
Supplement: Additional data file 5 — Presented is a table of major allele frequencies for biallelic SNPs surveyed across Lake Malawi cichlid populations and species. [file gb-2008-9-7-r113-S5.pdf]

Supplementary Table 5. Major allele frequency for biallelic SNPs surveyed across Lake Malawi cichlid populations and species. The first ten loci represent positive controls as explained in the text. Two SNPs were predicted and genotyped in sws2b; genotypes were in perfect linkage so only one is shown here.

**snp/pop**	***aim1***	***mitf***	***ednrb***	***rhodopsin***	***sws1***	***sws2a***	***lws***	***dec1_1***	***dec1_3***	***ip3r* (EXON12)**
**Species**	**NA**	**NA**	**NA**	**NA**	**NA**	**NA**	**NA**	**NA**	**NA**	**NA**
**All MZ**	0.73	0.989	0.8	0.48	0.95	0.82	0.65	1	0.094	0.771
**All LF**	0.83	0.984	0.37	0.82	1	0.99	1	0.658	0.784	0.906
**F_{ST} (within MZ)**	**0.17**	**0.05**	**-0.004**	**0.733**	**0.572**	**0.114**	**0.514**	**NA**	**0.02**	**0.336**
**F_{ST} (within LF)**	**0.172**	**0.023**	**0.736**	**0.853**	**NA**	**-0.006**	**NA**	**0.599**	**0.667**	**0.066**
**F_{ST} (MZ v LF)**	**0.028**	**-0.004**	**0.303**	**0.2**	**0.0444**	**0.153**	**0.358**	**0.337**	**0.65**	**0.059**
**All mbuna (25 sp.)**	0.7	1	0.63	0.49	0.86	0.73	0.92	0.854	0.383	0.778
**All others (52 sp.)**	0.992	0.73	0.042	0.08	0.71	0.044	0.792	1	0.960	0.967
**F_{ST} (Mbuna v nonMbuna)**	**0.179**	**0.358**	**0.485**	**0.424**	**0.227**	**0.811**	**0.005**	**0.106**	**0.502**	**0.144**

**snp/pop**	***sws2b***	**snp14 (*csrp1*)**	***sema 3c* (Exon 12)**	***sema 3f* (snp32)**	**snp10**	**snp13**	**snp19**	**snp21**	**snp22**	**snp24**
**Species**	**NA**	**LF/MZ**	**LF/MZ**	**MA/MZ**	**CC/RE**	**MZ/RE**	**LF/RE**	**CC/MZ**	**LF/RE**	**CC/LF**
**All MZ**	0.989	0.946	0.94	0.62	1	0.978	0.76	0.55	0.91	0.82
**All LF**	0.96	0.04	0.9	0.26	1	0.962	0.27	0.96	0.22	0.85
**F_{ST} (within MZ)**	**-0.01**	**0.033**	**0.135**	**0.195**	**NA**	**0.009**	**0.115**	**0.218**	**0.23**	**-0.002**
**F_{ST} (within LF)**	**0.233**	**0.076**	**0.179**	**0.557**	**NA**	**0.059**	**0.474**	**0.175**	**0.286**	**0.216**
**F_{ST} (MZ v LF)**	**0.009**	**0.893**	**-0.004**	**0.348**	**NA**	**-0.005**	**0.366**	**0.356**	**0.643**	**-0.002**
**All mbuna (25 sp.)**	0.85	0.69	0.86	0.54	1	0.9	0.62	0.91	0.92	0.43
**All others (52 sp.)**	0.812	0.988	0.87	0.02	0.836	1	0.55	0.992	1	0.21
**F_{ST} (Mbuna v nonMbuna)**	**0.097**	**0.382**	**0.009**	**0.363**	**0.249**	**0.034**	**-0.004**	**0.15**	**0.277**	**0.443**

snp/pop	snp25	snp27	snp30	snp33	snp44	Aln100099_882	Aln100200_1799	Aln100260_1099	Aln100281_1741	Aln100340_830
**Species**	**LF/RE**	**CC/LF**	**MZ/RE**	**LF/MA/MZ**	**MZ/RE**	**CC/LF**	**LF/MA**	**CC/MZ**	**CC/LF**	**MA/MZ**
**All MZ**	0.97	1	0.66	0.83	0.9	0.995	1	0.844	0.021	1
**All LF**	0.88	0.93	0.89	0.15	0.97	0.411	0.642	0.927	0.891	1
**F_{ST} (within MZ)**	**0.052**	**NA**	**0.218**	**0.174**	**0.112**	**-0.003**	**NA**	**0.114**	**0.023**	**NA**
**F_{ST} (within LF)**	**0.337**	**0.443**	**0.439**	**0.21**	**0.066**	**0.35**	**0.354**	**0.197**	**0.19**	**NA**
**F_{ST} (MZ v LF)**	**0.051**	**0.068**	**0.141**	**0.613**	**0.031**	**0.574**	**0.352**	**0.028**	**0.863**	**NA**
**All mbuna (25 sp.)**	0.94	0.992	0.86	0.62	0.92	0.772	0.861	0.896	0.383	0.984
**All others (52 sp.)**	0.98	1	0.05	0.984	0.992	1	1	0.996	0.887	1
**F_{ST} (Mbuna v nonMbuna)**	**0.031**	**0.017**	**0.73**	**0.392**	**0.048**	**0.171**	**0.1**	**0.074**	**0.446**	**0.005**

snp/pop	Aln100348_1026	Aln100388_766	Aln100532_2174	Aln100617_726	Aln100645_802	Aln100674_885	Aln100815_1005	Aln100949_327	Aln100993_1029	Aln101062_501
**Species**	**CC/LF**	**LF/MA**	**LF/RE**	**MA/RE**	**MA/RE**	**LF/RE**	**MA/RE**	**CC/MZ/RE**	**LF/RE**	**CC/MA/MZ**
**All MZ**	0.408	0.78	1	0.770	0.941	1	0.989	1	0.957	1
**All LF**	0.037	0.7	1	1	1	1	0.626	1	0.777	1
**F_{ST} (within MZ)**	**0.25**	**0.14**	**NA**	**0.247**	**0.178**	**NA**	**0.001**	**NA**	**0.118**	**NA**
**F_{ST} (within LF)**	**0.212**	**0.309**	**NA**	**NA**	**NA**	**NA**	**0.455**	**NA**	**0.179**	**NA**
**F_{ST} (MZ v LF)**	**0.328**	**0.008**	**NA**	**0.231**	**0.052**	**NA**	**0.344**	**NA**	**0.126**	**NA**
**All mbuna (25 sp.)**	0.285	0.62	0.998	0.891	0.948	1	0.803	1	0.853	1
**All others (52 sp.)**	0.951	0.984	0.972	1	0.966	0.960	0.627	0.826	0.996	0.614
**F_{ST} (Mbuna v nonMbuna)**	**0.591**	**0.227**	**0.03**	**0.078**	**-0.002**	**0.059**	**0.083**	**0.245**	**0.105**	**0.518**

snp/pop	Aln101106_1089	Aln101178_1198	Aln101315_386	Aln101457_685	Aln101889_931	Aln102022_261	Aln102321_608	Aln102504_1609	Aln103534_280	Aln104280_597
**Species**	**LF/MZ**	**MZ/RE**	**LF/MZ**	**CC/MA**	**CC/MZ**	**LF/MZ**	**CC/MA/RE**	**MA/RE**	**MA/RE**	**CC/MZ**
**All MZ**	0.903	0.677	0.75	1	0.891	0.777	1	1	0.99	0.963
**All LF**	0	0.810	1	1	0.973	0.552	1	1	1	1
**F_{ST} (within MZ)**	**0.171**	**0.132**	**0.019**	**NA**	**0.065**	**0.034**	**NA**	**NA**	**0.004**	**0.14**
**F_{ST} (within LF)**	**NA**	**0.139**	**NA**	**NA**	**0.271**	**0.5**	**NA**	**NA**	**NA**	**NA**
**F_{ST} (MZ v LF)**	**0.904**	**0.037**	**0.253**	**NA**	**0.047**	**0.097**	**NA**	**NA**	**0**	**0.033**
**All mbuna (25 sp.)**	0.534	0.701	0.9	1	0.925	0.704	1	1	0.79	0.979
**All others (52 sp.)**	0.988	0.980	0.992	0.963	0.976	0.740	0.967	0.066	0.03	0.996
**F_{ST} (Mbuna v nonMbuna)**	**0.388**	**0.222**	**0.085**	**0.057**	**0.018**	**-0.002**	**0.048**	**0.984**	**0.919**	**0.006**

snp/pop	Aln105886_636	Aln107484_583	Aln108541_1573	Aln110805_783	Aln110861_250	Aln111543_577	Aln112832_520	Aln114498_746	Aln115071_1230	Aln115522_691
**Species**	**CC/MA**	**CC/RE**	**CC/MA**	**CC/MA**	**CC/RE**	**LF/RE**	**LF/MA**	**CC/RE**	**MA/RE**	**LF/MA**
**All MZ**	0.99	1	1	0.43	1	0.647	0.995	1	1	1
**All LF**	0.99	0.989	1	0.51	1	0.286	1	1	1	0.49
**F_{ST} (within MZ)**	**-0.005**	**NA**	**NA**	**0.249**	**NA**	**0.088**	**-0.003**	**NA**	**NA**	**NA**
**F_{ST} (within LF)**	**0.057**	**-0.012**	**NA**	**0.238**	**NA**	**0.49**	**NA**	**NA**	**NA**	**0.266**
**F_{ST} (MZ v LF)**	**-0.005**	**0.005**	**NA**	**0.007**	**NA**	**0.225**	**0**	**NA**	**NA**	**0.525**
**All mbuna (25 sp.)**	0.97	1	1	0.55	1	0.490	0.996	1	0.998	0.99
**All others (52 sp.)**	0.75	0.95	0.832	0.912	0.832	0.984	0.956	0.960	0.617	1
**F_{ST} (Mbuna v nonMbuna)**	**0.408**	**0.054**	**0.252**	**0.333**	**0.248**	**0.406**	**0.043**	**0.06**	**0.503**	**0.145**

snp/pop	Aln116023_698	Aln116120_573	Aln117472_1109	Aln118796_231	Aln119531_730	Aln119718_628	Aln121593_508
**Species**	**CC/MZ**	**LF/MA**	**MA/MZ**	**MA/RE**	**MZ/RE**	**CC/MA**	**CC/RE**
**All MZ**	0.590	1	0.947	1	0.936	0.989	0.819
**All LF**	0.865	1	0.958	1	1	0.77	0.964
**F_{ST} (within MZ)**	**0.139**	**NA**	**0.058**	**NA**	**0.473**	**-0.005**	**0.327**
**F_{ST} (within LF)**	**0.491**	**NA**	**0.045**	**NA**	**NA**	**0.36**	**0.264**
**F_{ST} (MZ v LF)**	**0.168**	**NA**	**-0.004**	**NA**	**0.057**	**0.201**	**0.097**
**All mbuna (25 sp.)**	0.727	0.992	0.906	1	0.977	0.94	0.871
**All others (52 sp.)**	0.992	0.984	0.989	0.989	1	1	0.520
**F_{ST} (Mbuna v nonMbuna)**	**0.21**	**-0.002**	**0.061**	**0.02**	**0.013**	**0.074**	**0.293**
